# Context-Dependent *Egr1* Expression in the Avian Hippocampus

**DOI:** 10.1371/journal.pone.0164333

**Published:** 2016-10-07

**Authors:** Stephanie L. Grella, Mélanie F. Guigueno, David J. White, David F. Sherry, Diano F. Marrone

**Affiliations:** 1 Department of Psychology, Wilfrid Laurier University, Waterloo, ON, Canada, N2L 3C5; 2 Department of Biology, University of Western Ontario, London, ON, Canada, N6A 3K7; 3 Department of Psychology, University of Western Ontario, London, ON, Canada, N6A 3K7; 4 McKnight Brain Institute, University of Arizona, Tucson, AZ, United States of America, 85719; University of Lethbridge, CANADA

## Abstract

In mammals, episodic memory and spatial cognition involve context-specific recruitment of unique ensembles in the hippocampal formation (HF). Despite their capacity for sophisticated spatial (e.g., for migration) and episodic-like (e.g., for food-caching) memory, the mechanisms underlying contextual representation in birds is not well understood. Here we demonstrate environment-specific *Egr1* expression as male brown-headed cowbirds (*Molothrus ater*) navigate environments for food reward, showing that the avian HF, like its mammalian counterpart, recruits distinct neuronal ensembles to represent different contexts.

## Introduction

In mammals, the hippocampal formation (HF) critically mediates spatial cognition via the creation of internal “maps” of the environment generated by place cells [1]. Importantly, these cells are thought to also mediate episodic memory using comparable mechanisms (e.g., [2]). Like mammals, many bird species exhibit sophisticated spatial (e.g., [3]) and episodic-like (e.g., [4]) memory, and this robust repertoire has motivated many attempts to characterize the homology between the avian and mammalian HF (reviewed in [5,6,7]). For instance, tract tracing has demonstrated that both mammals and birds have similar sets of inputs to and outputs from the HF. Moreover, lesions to the HF produce comparable deficits in spatial processing [6,8].

At the cellular level, avian HF neurons exhibit place-like firing properties in at least some conditions [9,10,11]. The degree to which these spatially-tuned cells form coherent context-dependent place maps, however, remains unknown. This issue is important given the differences in intrinsic connectivity of the HF between mammals and birds. Although the subdivisions of the avian HF remain a matter of debate [12,13], this structure lacks many of the defining features of its mammalian counterpart, including clear trilaminar structure, a dominantly unidirectional tri-synaptic loop, and perhaps even a homologue to the dentate gyrus [7]. Because each sub-structure is thought to provide a unique contribution to sculpting spatial representations [14,15], anatomical differences may translate into dramatic differences in place map formation.

**Fig 1:**
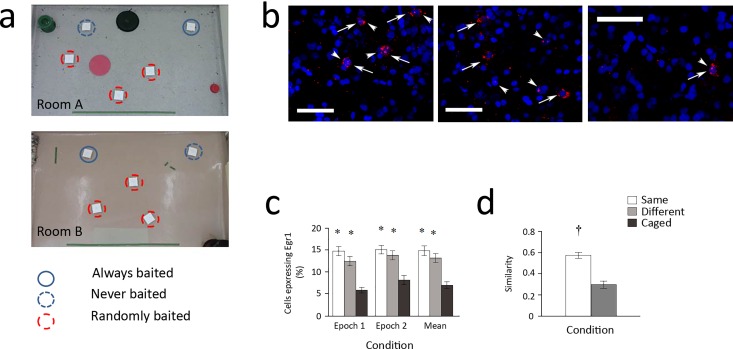
*Egr1* expression in the cowbird hippocampus is context-dependent. (a) Two rooms with different visual cues (floor and wall) were available for foraging, each with a distinctive arrangement of cups (birds displaced paper lid to obtain food reward). (b) Sample confocal images (scale bar = 50 μm) showing intranuclear foci signal (*Egr1* transcription 0–5 min before sacrifice, short arrows), and cytoplasmic signal (*Egr1* transcription 25–30 min before sacrifice, long arrows). (c) Foraging cowbirds expressed *Egr1* in significantly more cells than birds that remained in the home cage (Caged: dark grey). (d) Similarity scores show that the proportion of cells that repeatedly expressed *Egr1* across both explorations was significantly higher in birds that explored the same room twice (Same: white) relative to birds that explored different rooms (Different: light grey). Data are means ±SEM (* = p <0.05 vs. Caged; † = p< 0.05 vs. different).

To determine if context-specific populations of cells are recruited in the avian HF, we examined the transcription of *Egr1* in brown-headed cowbirds as they explored environments in search of food reward (Fig 1A). This was done by adapting cellular compartmental analysis of temporal fluorescence *in situ* hybridization (catFISH), a technique developed in mammals [16]. This technique relies on visualizing expression of immediate-early genes, including *Egr1*, which is tightly coupled to neuronal activity, is a critical mediator of plasticity [17], and reliably reports place cell activity [18]. By determining the compartmental expression (nucleic or cytoplasmic) of *Egr1*, we show that the avian HF produces context-dependent patterns of *Egr1* transcription that are comparable to those observed in the mammalian hippocampus.

## Materials and Methods

### Subjects

Fourteen adult male brown-headed cowbirds (*Molothrus ater*) were individually housed in a temperature- and humidity-controlled colony room at the Advanced Facility for Avian Research (University of Western Ontario) on a 12:12 h light/dark cycle (lights on at 07:30). Water was accessible *ad libitum* and food restricted to maintain 85% of their *ad libitum* body weight in captivity. The University of Western Ontario Animal Users’ Subcommittee under Canadian Council on Animal Care guidelines approved all procedures.

### Apparatus

Two distinct contexts, each containing 5 food cups on the floor, were used (Fig 1A). In each room (2.4m x 3.6m), distinct visual cues were placed on the walls and floors, and the cups were arranged in a distinct geometric pattern in part of the room (2.4m x 1.6m).

Room A had a gray-colored epoxy-coated concrete floor flecked with black and white. A stainless steel chair rail (10 cm width) ran the full length of three walls at a height of 80 cm above the floor. The steel door to the room was light green. Glossy colored photos and large sheets of brown paper (1 m X 6 m) were attached to the walls of the room. On the floor were an inverted dark green plastic pail (20 cm diameter, 20 cm in height), two circular rubber mats approximately 30 cm in diameter (one red, one black), and an inverted red food cup (15 cm diameter).

Room B had a tan-colored epoxy-coated non-flecked soft surface floor and no chair rail. A sloped channel 1.9 m wide ran from the left to the right wall, increasing in depth from 5 cm on the left to 15 cm on the right. The steel door to this room was gray. There was a 40 cm X 40 cm stainless steel panel in the wall to the left of the door 70 cm above the floor and two 45 cm X 45 cm stainless steel panels at floor level in the left and right walls. A green extension cord that powered a camera attached to the ceiling traveled down the left wall of this room to a power outlet. An inverted black plastic pail (30 cm diameter, 30 cm in height) covered a drain at the right end of the floor channel and a rolled fabric mat (10 cm diameter, 45 cm in length) was placed against the floor-level stainless steel panel in the left wall. Room A was 3 m from the birds’ holding rooms, and room B was 15 m away in the same direction along the same hallway.

### Behavioral training

In order to establish that cowbirds are able to discriminate the two environments used in this experiment, the birds were tested on their ability to remember the locations of food cups baited with bird seed in each of the two rooms (Fig 1A).

For each trial, two of the 5 cups were baited: a consistently baited cup and a randomly baited cup. The consistently baited cup remained the same on every trial. In Room A, this cup was in the NE corner, and in Room B in the NW corner of the array. Search and accurate choice of the consistently baited cup was recorded to determine whether the birds recognized which of the two rooms they were in. Additionally, a randomly selected cup in each room was also baited on each trial. The purpose of the randomly baited cup was to encourage continued spatial search and context exploration by the bird in each room. All cups were covered with a cardstock lid that the birds had to displace in order to see the contents of the cup. Seeds were added and removed from the unbaited cups before each trial in order to ensure the birds did not use olfactory cues to locate the baited cup. For each trial, the birds entered the room and searched the array of cups for food by displacing each card. A cup was considered “searched” when the bird displaced the lid from the cup. All trials were recorded with an overhead camera. Each room was divided into 6 equally sized grids for determination of the spatial occupancy of birds during the final testing sessions.

Training began with 2 days of habituation where birds visited both rooms for 30 min each day with all cups baited and uncovered. Over the course of approximately 2 months of training, the time spent in each room was gradually reduced to 5 min, and the number of cups baited was gradually reduced to 2, and cups were covered with cardstock paper lids. A complete trial consisted of 5 min in Room A followed by 20 min in the home cage followed by 5 min in Room B. The order of experience in the two rooms varied on successive trials and was pseudo-randomly determined. All birds received one trial per day. During the 20 min home cage interval, birds were not given access to food. Successful task performance consisted of finding the food reward in the consistently baited cup within the first 3 search attempts. A cup was considered “searched” when the cardstock lid covering it was displaced. After finding this reward, birds were permitted to continue to search for the food reward in the randomly baited cup (if they had not already found it). Birds were trained until they reached a criterion of 3 consecutive successful trials. They were given a time limit of 5 min to complete the task. If they completed the task sooner, the trial was terminated. Birds were tested on the day following the last successful trial to reach criterion.

### Test day

Cowbirds engaged in two 5-min episodes of exploration, separated by 25 min in the home cage. There were 5 groups. Birds were either placed in rooms A/A (n = 4), A/B (n = 3), B/A (n = 3), B/B (n = 1), or left undisturbed in the home cage (CC, n = 3). No cups were baited on the final day. This was done in order to ensure that the birds would traverse the environment for the full duration of the trial. The absence of reward is unlikely to affect *Egr1* expression because similar levels and patterns of *Egr1* expression are obtained in the presence [19] and absence [18,20] of rewards.

### Brain extraction and tissue preparation

Within a period of 3 min following the end of the trial, birds were anesthetized with isoflurane, transported to a procedure room, decapitated, and the brains rapidly extracted and flash frozen in 2-methylbutane (Sigma Aldrich) immersed in dry ice/ethanol. Brains were packed in dry ice and transported to Wilfrid Laurier University for processing. Coronal sections (20 μm) were cut using a CM3050 cryostat (Leica), thaw-mounted onto slides coated with 3-triethoxysilylpropylamine (VWR), dried, and stored at -80°C.

### Fluorescence in situ hybridization

Fluorescence *in situ* hybridization was performed as previously described [16,18], with the exception of the riboprobe used. Briefly, riboprobe was synthesized by reverse transcription from a commercially available 807-base EST plasmid of the chicken (*Gallus gallus*) EGR1 (Source BioScience, GenBank accession number BU273177) using a transcription kit (MaxiScript; Ambion) and RNA labeling mix (Roche Molecular Diagnostics), and verified by electrophoresis. Slides were warmed to -20°C overnight and to room temperature (RT) 1 hr before processing. They were fixed in 4% formaldehyde (5 min), washed in 2x saline-sodium citrate (SSC) for 2 min, and treated with 0.5% acetic anhydride for 10 min. Next, they were rinsed in deionized water, placed in a methanol/acetone (1:1) solution (5 min), and in 2x SSC (5 min). Slides were incubated with pre-hybridization buffer (Sigma-Aldrich) for 1hr at RT and then overnight (16–18 hrs) at 56°C with riboprobe mixed in hybridization buffer (100ng/slide).

The tissue was then treated with a series of SSC washes followed by RNase A (10 mg/ml) at 37°C for 15 min. Endogenous peroxidases were quenched with 2% H_2_O_2_ (in 1xSSC) for 15 min. The tissue was blocked with TSA blocking buffer (Perkin Elmer) containing normal sheep serum (0.5%), and incubated with anti-digoxigenin horseradish peroxidase (HRP) antibody (Roche Molecular Diagnostics) in TSA blocking buffer (1:400) for 2 hrs at RT. Slides were washed in 0.1M Tris-buffered saline with 0.05% Tween-20 and HRP antibody conjugates were detected using CY3 (TSA kit, Perkin Elmer). Nuclei were counter-stained with 4',6-diamidino-2-phenylindole (DAPI, Sigma-Aldrich), before slides were mounted in buffered glycerol containing n-propyl gallate as an anti-fade additive [21], coverslipped, and sealed with nail polish.

### Image acquisition and analysis

Images were collected from coronal sections of the HF (range: ~1mm lateral to midline, 3.33–3.87 mm rostral to the Y-point, see [22]), using an Olympus FV1000 confocal microscope at 40x magnification. For each animal, 3 slides each containing one coronal section were chosen from this range. For each slide 2–4 images were taken. Each image collected was a combination of two z-stacks (~1.0um optical thickness, step size 0.8um). For each slide, acquisition parameters were kept constant. The median 20% of HF cells in each stack was quantified using MetaMorph software (Molecular Devices). Neurons and glial cells were differentiated, and neurons were classified as *Egr1*-negative, *Egr1*-positive within the nucleus, *Egr1*-positive within the cytoplasm, and *Egr1*-positive within both the nucleus and the cytoplasm. An average of 1879 cells (standard deviation = 960.50) was counted per animal. Based on the time course of *Egr1* transcription and translation, it is known that cells expressing *Egr1* in the cytoplasm were engaged in *Egr1* transcription 25–30 min prior to the sacrifice of the animal (i.e., during the first context exposure), while cells expressing *Egr1* in the nucleus were engaged in transcription 5 min before sacrifice (i.e., during the second context exposure).

### Statistical analysis

Differences in the pattern of *Egr1* expression were analyzed as previously described [18,19]. Briefly, animals in the A/A and B/B groups were pooled to create the group 'Same' (n = 5) and animals in the A/B and B/A groups were pooled to create the group 'Different' (n = 6). Pooling yields sample sizes within the typical range of the samples used in comparable rodent studies [16,23–28]. The use of relatively small groups for this type of analysis is common largely because the effects being studied (i.e., remapping-related Egr1 expression) are quite large [16,23–28], and so do not typically require a large sample in order to obtain sufficient power.

The percentage of cells expressing *Egr1* within each behavioral epoch was calculated. The estimate of the fraction of the total cell population that is active during epoch 1 (E1) includes cells containing *Egr1* in both cellular compartments as well as cells containing *Egr1* solely in the cytoplasm. The estimate of the fraction of the total cell population that is active during epoch 2 (E2) includes cells containing *Egr1* in both cellular compartments as well as cells containing *Egr1* solely within the nucleus.

In addition to quantifying the absolute number of cells expressing *Egr1* during each epoch, the overlap in the representations (i.e., the probability that the same cell expressed Egr1 in response to both environments) was quantified using the similarity score used by Vazdarjanova and Guzowski [23]. The similarity score is derived from E1 and E2 (described above) as follows: similarity = (D -p(E1E2))/(L–p(E1E2)). In this equation, D = (fraction of cells *Egr3+* in both cellular compartments), while p(E1E2) = E1 × E2 (joint probability). Finally, L = the smaller of E1 and E2. This term normalizes the similarity score such that a perfect overlap (i.e., 100% of the cells expressing *Egr1* during E1 also express *Egr1* during E2) is 1, and overlap equal to random chance is 0.

A one-way analysis of variance compared total cellular *Egr1* expression during each behavioral epoch. Pairwise comparisons were then conducted using an independent *t*-test with Bonferroni correction. Data are presented as means ± standard error of the mean.

## Results

### Cowbirds can discriminate the two environments

Cowbirds were able to discriminate the two environments. By the final day of training, cowbirds displaced the lid of the consistently baited cup first on 96.3% (±2.5%) of trials. Birds generally walked on the floor but occasionally made hops or short flights. Of the 6 zones in each room, birds entered on average 4.14 zones on each trial (mean Room A = 4.14; mean Room B = 4.13). Total distance moved ranged from 2.83 to 14.68 m in Room A (mean = 5.82 m) and from 4.43 to 11.17 m in Room B (mean = 6.76 m). Mean rates of movement were 2.56 cm/s in Room A and 2.27 cm/s in Room B.

### *Egr1* transcription in the avian hippocampus is context-dependent

Exploration induced a robust increase in *Egr1* expression in the avian HF (main effect: *F*_2, 11_ = 13.568; *p* = 0.001). In the HF of exploring cowbirds, *Egr1* was observed in approximately15% of cells, somewhat lower than either the histological [29] or electrophysiological [24] estimates of cellular recruitment in rodents (Fig 1C).

Most importantly, the number of environments explored significantly altered the pattern of *Egr1* expression (t_9_ = 5.733; *p* < 0.001). Cowbirds that explored the same context twice demonstrated a significantly higher similarity score relative to birds that explored two different contexts (Fig 1D). These data demonstrate that, as in mammals, the pattern of *Egr1* expression in the avian HF is contextually mediated.

## Discussion

The current results demonstrate that the pattern of *Egr1* expression (and by extension neuronal firing) in the HF of cowbirds as they navigate space is remarkably consistent with mammalian data under comparable circumstances. As a result, these data not only provide the first demonstration of context-specific gene expression in the avian HF, but also corroborate electrophysiological evidence for the existence of “place-like” cells in the avian HF tuned to specific stable goal locations [9–11]. This finding provides avenues for comparative testing of contemporary hypotheses about hippocampus-dependent cognition. Although the current experimental protocol focuses exclusively on the spatial dimension of hippocampal processing, the current data open the door to examine *Egr1* expression patterns during the processing of other forms of information. Such comparisons are particularly intriguing given the wealth of compelling evidence for episodic-like memory in avian species (e.g., [30–32]), as well as recent mammalian data showing processing of non-spatial information, such as time (e.g., [33]) may use a common computational framework with space (e.g., [34]) in the mammalian HF.

These lines of investigation become particularly important when considering that the avian HF lacks (at least unequivocally) many of the defining features of the mammalian hippocampus, such as clear trilaminar structure, a dominantly unidirectional tri-synaptic loop, and perhaps even a homologue to the dentate gyrus [7]. These observations indicate that birds provide a potentially important model to test ideas about the necessity and sufficiency of features of hippocampal structure in generating refined contextual maps.

In addition, there has been a resurgence in interest about how space is represented in the vertical dimension [35], inspired at least in part by recent characterization of the bat HF [36]. Comparison of spatial maps of flying and non-flying birds alongside flying and non-flying mammals can provide powerful convergent evidence regarding if and how the spatial navigation system is tailored for flight in these animals.
